# Quality Characteristics and Functional and Antioxidant Capacities of Algae-Fortified Fish Burgers Prepared from Common Barbel (*Barbus barbus*)

**DOI:** 10.1155/2019/2907542

**Published:** 2019-10-09

**Authors:** Faiez Hentati, Mohamed Barkallah, Ali Ben Atitallah, Mouna Dammak, Ibtihel Louati, Guillaume Pierre, Imen Fendri, Hamadi Attia, Philippe Michaud, Slim Abdelkafi

**Affiliations:** ^1^Unité de Biotechnologie des Algues, Biological Engineering Department, National Engineering School of Sfax, University of Sfax, 3038 Sfax, Tunisia; ^2^Université Clermont Auvergne, CNRS, SIGMA Clermont, Institut Pascal, F-63000 Clermont-Ferrand, France; ^3^Laboratoire de Génie Enzymatique et de Microbiologie, University of Sfax, National Engineering School of Sfax, B.P. 1173-3038 Sfax, Tunisia; ^4^Laboratory of Plant Biotechnology Applied to the Improvement of Cultures, Faculty of Sciences of Sfax, B.P. 1171, No. 3000, University of Sfax, 3029 Sfax, Tunisia; ^5^Laboratory of Analysis Valorization and Food Safety, National Engineering School of Sfax, University of Sfax, 3038 Sfax, Tunisia

## Abstract

**Introduction:**

Algae have been used as natural ingredients to produce new canned fish burgers prepared from minced flesh of common barbel. In this research, the impact of the addition of* Cystoseira compressa *and* Jania adhaerens* at concentrations of 0.5, 1, and 1.5% w/v on the texture and sensory characteristics of fish burgers were investigated.

**Results:**

Compared to controls, fish burgers containing 1% algae had better texture and sensory properties (*P* < 0.05). Also, these burger formulations had higher water and oil holding capacities as well as swelling ability, due to the important polysaccharides and dietary fibers contents of algae. In addition, algae-supplemented burgers were characterized as having low L^⁎^, a^⁎^, and b^⁎^ values, which made the color appear to be paler. Thanks to their high richness in pigments (chlorophylls and carotenoids) and polysaccharides, algae considerably enhance the antioxidant activities of the new ready-to-eat fish burgers. So,* Cystoseira compressa *and* Jania adhaerens* could be used as nutritious additives to produce new fish-based products.

## 1. Background

Fishing in Tunisia has mostly exploited the benthic stocks which are actually in optimal exploitation state or even overexploitation. This condition has incited the Tunisian authorities to search effective solutions such as the development of aquaculture and the recovery of untapped fish. As a result, the idea of exploiting freshwater fish species (Tilapia of the Nile, common carp (*Cyprinus Carpio*), and common barbel (*Barbus barbus*)) in intensive farming has emerged. The common barbel is a freshwater fish species that belongs to the* Cyprinidae* family and exists in many Tunisian dams thanks to its good adaptation to the environment. His flesh is rich in *ω*3-series polyunsaturated fatty acids with long chains such as eicosapentaenoic acid (EPA) and docosahexaenoic acid (DHA), fat-soluble vitamins (A, D, and especially E), water-soluble vitamins (B12 and especially B6) and low levels of cholesterol and saturated fats [1]. All these compounds are beneficial to people's health and they are necessary for the growth and protection of the human body [1]. The presence of intramuscular bones in the flesh of common barbel is the cause of its low consumption by the Tunisians. Hence, it is very substantial to create and produce new fish-based products from these underutilized fish species so as to enhance their consumer acceptability.

Despite their health benefits, fish-based products are not generally considered as a primary source of bioactive compounds (polysaccharides, pigments…) [2]. In the agro-alimentary industries, various synthetic additives have been popularly used for the aims of coloring, fortifying, and extending the shelf-life of the marketed products [3, 4]. Nevertheless, many recent studies have indicated that the unreasonable consumption of artificial additives is related to many health problems [5, 6]. Thus, the need to use natural food fortifiers has pushed nutrition experts to generate supplements from natural resources that might be suitable for food products [4, 7].

Recently, the consumption of marine products such algae (*C. compressa* and* J. adhaerens*) has gradually increased for the gradual awareness of the close relationship between diet and health [8]. As a result, many food products containing algae have been marketed. Indeed, algae are an interesting source of natural antioxidants and antimicrobials agents which have been successfully used in some foods such as porcine [9], beef [10], chicken [11], fish and seafood [12, 13] products. They can turn food more functional with high added value and so, they can guarantee the consumer's satisfaction. To the best of our knowledge, studies regarding the use of algae in processed fish products are lacking.

In this respect, this study has two principal objectives. The first one is to produce new eatable fish burgers prepared from minced flesh of common barbel and fortified with algae (*Cystoseira compressa* and* Jania adhaerens*), which are rich in bioactive compounds (pigments and polysaccharides). The second one is to assess the beneficial effects of algae on the sensorial, textural, physicochemical, microbiological, functional, and antioxidant properties of these fish products.

## 2. Materials and Methods

### 2.1. Algae

In 2016,* J. adhaerens *and* C. compressa* were harvested from Tabarka (Governorate of Jendouba, Tunisia) and Kerkennah island (Governorate of Sfax, Tunisia), respectively. After cutting them, they were washed with fresh water, sun-dried at ambient temperature, milled, treated with a sieve of 0.2 mm mesh and stored in sealed bags under room temperature. The algae powders obtained were analyzed for their moisture, protein, ash and fat according to the AOAC official method (Method 993.21.) [14]. Total carbohydrate and uronic acid concentrations were evaluated after acid hydrolysis (H_2_SO_4_ (96%), 1h, 30°C) by the phenol-sulfuric acid method [15] and m-hydroxydiphenyl (MHDP) assay [16], respectively. Functional and chemical composition, including water holding capacity, oil absorption capacity, total dietary fiber and pigments were determined using the same methods as described in the coming parts (Sections 2.5 and 2.6).

### 2.2. Fish Collection

Freshwater fish (common barbel,* Barbus barbus*) were sampled in January 2017 from the reservoir of Sidi Salem (Governorate of Beja) by professional fishermen, using gill nets of mesh size equal to 80 mm. The fish were between 40 and 55 cm long and their weight ranged from 0.7 to 2.5 kg. Next, they were stored in isotherm ice polyethylene boxes (~0°C) and 1 h after they were immediately transported to the pilot unit of fish processing of Tabarka in order to test their freshness. The samples were then washed, weighed, scaled, eviscerated and fileted so as to obtain clean fish fillets. After being cut into small pieces, the skinless fish filets were minced during 2-3 min in a blender (Robot Coupe USA Inc., Ridgeland, MS, USA) and kept at -20°C for 48 h.

### 2.3. Formulation and Production of Fish Burger

All the stages of the fish burgers processing were performed in the pilot fish processing unit of Tabarka (Jendouba, Tabarka, Tunisia) in collaboration with the Interprofessional Grouping of the Fishery Products (GIPP, Tunis, Tunisia). After being thawed overnight in the refrigerator, the minced meat was thoroughly mixed with salt (2%, w/w), cornstarch (Spipa, Tunis, Tunisia) (1%, w/w) and with different concentrations (0.5, 1 and 1.5%, w/w) of algae (*C. compressa* and* J. adhaerens*) powders. A combination of* C. compressa/J. adhaerens* (proportions of 1/1, w/w) was also tested depending to their preliminary results.

Then, the shaping of fish burgers was then carried out using a commercial burger maker (Hamburger MV NEW model, Food Tech Srl, Bologna, Italy) in order to get disc burger pieces which 6 cm wide and 1.5 cm thick and which weigh 100 g. Algae-free burgers were used as controls. Burgers were separately placed in a well-washed and wiped metal tin can that was 8.54 cm wide and 3.7 cm high, immediately soaked in sunflower oil (Safi, Ben Arous, Tunisia) and then packaged by a crimping machine (Seamer Semiautomatica, MOD.AGM, S-Bologna, Italy) in order to ensure complete sealing. Finally, the cans were washed, for the purpose of removing the excess of oil, and sterilized in a retort at 120°C for 40 min [17]. They were stored in an alimentary refrigerator at 4°C for further analyses (8 months).

### 2.4. Nutritional Properties of Algae Enriched Fish Burgers

#### 2.4.1. Dry Matter, Moisture and Ash Content

Dry matter (DM) and ash were determined according to AOAC [14] method. DM was determined by drying samples at 105°C for 24 h in an air oven (Thermoline Scientific, Australia). The mineral content was quantified after heating the samples at 550°C for 4 h in an electric muffle furnace (Labec Laboratory Pty Ltd., Marrickville, NSW, Australia). This was expressed as percentage of ash in DW.

#### 2.4.2. Protein, Dietary Fiber and Lipid Content Analysis

The protein content was determined by the Kjeldhal assay according to AOAC International method [14] with a nitrogen conversion factor of 6.25 [18]. The total dietary fibers (TDF) were quantified by the nonenzymatic gravimetric method (AOAC Official Method 993.21.) based on the precipitation of fibers with ethanol [19]. The total lipids of burger were extracted and quantified according to the modified procedure of Bligh and Dyer [20] with chloroform, methanol, and water (2/1/1, v/v).

### 2.5. Chemical Composition and Antioxidant Potential of Algae Enriched Fish Burger

#### 2.5.1. Chlorophylls and Carotenoids Contents

The carotenoid and chlorophylls contents were determined by spectrophotometry as described by Lichtenthaler and Wellburn [21] and Kumar et al. [22] after adding 2 mL of ethanol (96%) to 0.2 g of samples and letting them incubate at 65°C for 30 min under a continuous stirring. After sonication (15 min, 60 W, 40 kHz) by using an ultrasonic bath (Bandelin Electronic, Berlin, Germany), the suspensions were centrifuged at 10,000 g for 5 min at 20°C. The concentrations of pigments in supernatants were measured at 666, 653 and 470 nm using the Equations (1), (2) and (3):

(1) $$\begin{array}{c} \left[Chl\;a\right]\;\left(mg/L\right)=15.65\times A_{666}-7.340\times A_{653} \end{array}$$

(2) $$\begin{array}{c} \left[Chl\;b\right]\;\left(mg/L\right)=27.05\times A_{653}-11.21\times A_{666} \end{array}$$

(3) $$\begin{array}{c} \left[Carotenoids\right]\;\left(mg/L\right)=\frac{\left(1000\times A_{470}-2.86\times \left[Chl\;a\right]-85.9\times \left[Chl\;b\right]\right)}{245} \end{array}$$

#### 2.5.2. FT-IR Spectroscopy

The absorption spectra of all burger formulations were obtained using Fourier Transform Infrared Spectroscopy (FTIR) (Agilent Technologies Spectrophotometer, Cory 630 FT-IR). For that, dried samples were deposited on accessory plate. The transmission spectra were obtained in the wave-number range of 600-4000 cm^−1^ at a resolution of 4 cm^−1^. For each sample, an average of 10 scans has been done. The acquisition and the processing of spectra have been carried out using the “Spectrum” software.

#### 2.5.3. Evaluation of Antioxidant Properties

The antiradical activity was measured using the synthetic radical DPPH (1,1-diphenyl-2-picrylhydrazyl) using the method of Bersuder et al. [23]. Approximately 10 mg of the sample was suspended in 0.5 mL of distilled water. After making up to 1.2 mL with 0.5 mL of absolute ethanol and 0.2 mL of DPPH (50 *μ*M in ethanol), the mixture was incubated for 30 min in the dark at room temperature. The absorbance was measured at 517 nm using the T70 UV–visible spectrophotometer (PG Instruments Ltd., Beijing, China). The control was done in the same manner, expect that distilled water was used instead of sample.

From the absorbance, % inhibition or % scavenging activity is calculated using the formula,

(4) $$\begin{array}{c} DPPH\;scavenging\;activity=\left(\frac{\left(OD\;control-OD\;sample\right)}{OD\;control}\right)\times 100 \end{array}$$

The ferric reducing antioxidant power (FRAP) of burger samples was determined using the method of Yildirim et al. [24]. Fish burgers were cut into small pieces (10 mg), immersed in 0.1 mL of distilled water and mixed with 2.5 mL of sodium phosphate buffer (NaHPO_4_, 0.2 M, pH 6.6) and 2.5 mL of 1% (w/v) potassium ferricyanide (K_3_Fe (CN)_6_). The mixtures were incubated for 30 min at 50°C. After incubation, 2.5 mL of 10% trichloroacetic acid (TCA) were added and the reaction mixtures were centrifuged at 10,000* g* for 10 min at 4°C. Finally, 2.5 mL of the upper layer solution was taken and mixed with 2.5 mL of distilled water and 0.5 mL of 0.1% ferric chloride. The procedure was carried out in triplicate and absorbance was measured at 700 nm.

### 2.6. Quality and Shelf-Life Measurements

#### 2.6.1. Physical Characteristics of Fish Burger

All the measurements were done during the first month after production and all measurements were carried out in triplicate. The pH was measured using a calibrated pH meter (Metrohm-744 pH meter, Heirisau Switzerland) equipped with a glass probe to ensure penetration into fish burger. The water activities (a_w_) of wet and dried products were determined using a calibrated SPRINT Novasina Thermoconstanter SPRINT TH500 (Axair Ltd., Pfäffikon, Switzerland) at 25°C. The equipment was previously calibrated according to the calibration procedure of the equipment manufacturer using the following salts: MgCl_2_, NaCl, BaCl_2_ and K_2_Cr_2_O_7_.

#### 2.6.2. Functional Properties of Fish Burger

The swelling capacity of fish burgers (SWC) was determined as described by Wong and Cheung [25] with slight modifications. Dried samples (200 mg) were placed in a 50 mL graduated glass cylinder. After making up the volume to 50 mL with distilled water, the mixtures were stirred for 2-3 min and then stored at ambient temperature for 24 h. The swelling volume was measured and expressed in mL of swollen sample per grams of dry weight (DW) of burger.

The water holding capacity (WHC) was measured according to Okezie and Bello [26] method. Sample (0.4 g) was mixed with 40 mL of distilled water in centrifuged tubes and stirred for 24 h at room temperature. After centrifugation (14,000 g, 4°C, 20 min), the supernatant was filtered through Whatman filter paper of porosity 1 (Whatman International Ltd., Maidstone, England) and the recovered volume (filtrate) was then measured. The difference between the initial volume and that of supernatant (WHC) was expressed as the weight of water held per gram of dry sample.

The oil holding capacity (OHC) was determined according to the method adapted from Wong and Cheung [25]. Three grams of dried fish burger were mixed with 8 g of corn oil (Safi, Ben Arous, Tunisia) and incubated at ambient temperature for a 30 min under continuous stirring. The mixture was then centrifuged at 2,500 g for 30 min at 20°C. The oil supernatant was then recovered and measured. OHC of burgers was expressed as grams of absorbed oil per gram of sample. All measurements were carried out in triplicates.

#### 2.6.3. Sensory Evaluation

The sensory evaluations of burgers were done according to the protocol proposed by Barkallah et al. [27] and Jridi et al. [28]. The attributes of burger samples were conducted by 32 panelists (22 female and 10 male) aged from 20 to 45 years, 7 days after the production of burgers. The tasting panel included agri-food engineers and biological researchers in the pilot unit of fish processing. Samples were distributed on polystyrene plates and presented to the panelists with three digit codes in a random order. Experiments were performed in a sensory evaluation room equipped with white light and controlled ventilation and water was served for perfectly cleaning the mouth between samples. Members of the sensory panel evaluated the fish burgers for taste, appearance, texture, color and odor based on a traditional 5 point-hedonic scale ranging from 1 (extremely disliked) to 5 (extremely liked) (1 = very bad, 2 = bad, 3 = neither bad nor good, 4 = good and 5 = very good) for each parameter [29]. A score of 4 was considered the threshold for acceptance of the fish burger.

#### 2.6.4. Color Analysis

Color evaluation of canned burger was made using a spectro-colorimeter (Konica Minolta, Chroma Meter, CR400, Japan). An average color value was expressed by measuring five different points of the same sample. The CIE-Lab color scale was used to measure the lightness (L^*∗*^), redness (+a^*∗*^) or greenery (−a^*∗*^) and yellowing (+b^*∗*^) or blue (−b^*∗*^). The instrument was calibrated using standard white plates with color coordinates of L^*∗*^ standard = 97.6, a^*∗*^ standard = 0.03 and b^*∗*^ standard = 1.73, supplied by Minolta. The color can also be expressed in polar (or cylindrical) coordinates L^*∗*^, C^*∗*^ and h^*∗*^, where L^*∗*^ is identical to that described previously, C^*∗*^ is the chroma or saturation index and h^*∗*^ is the color tint of the product. The following Equations ((5) and (6)) were used to convert the coordinates L^*∗*^  a^*∗*^  b^*∗*^ into cylindrical coordinates L^*∗*^  C^*∗*^  h^*∗*^:

(5) $$\begin{array}{c} C^{*}={\left(a^{{*}^{2}}+b^{{*}^{2}}\right)}^{0.5} \end{array}$$

(6) $$\begin{array}{c} h^{*}=arc\;tang\left(\frac{b^{*}}{a^{*}}\right) \end{array}$$

#### 2.6.5. Textural Analysis

Texture analysis of fish burgers stored for at least 24 h at 4°C was performed using a texture analyzer (Texture Analyseur, TA Plus, Lloyd Instruments, Bognor Regis, UK) equipped with a 1000 (N) load cell and 0.05 (N) detection range [30]. The test was applied directly on burgers which were 4 cm long and 4 cm wide using a 12-mm diameter analysis probe. The samples underwent a compression step of 50% of their original thickness in a double cycle with a rate of 40 mm/min. The pre-test speed and the target mode distance were set at 1.5 mm/s and 10 mm, respectively. The trigger force (trigger type: auto) was fixed at 5 g and the data acquisition rate was programmed at 200 points/s. The texture profile parameters such as cohesiveness, elasticity (mm) and firmness (N) were calculated from the resulting force-strain curves.

#### 2.6.6. Microbiological Analysis

The microbiological analyses of fish burger were examined throughout the storage period [31, 32]. The tests of* Escherichia coli*,* Enterobacteriaceae*, yeast, mold, coliforms and foodborne pathogens (*Salmonella* spp.,* Shigella* spp.,* Bacillus cereus*,* Listeria monocytogenes*, and* Staphylococcus aureus*) were performed using the standard microbiological methods for the analysis of ready-to-eat foods [33]. After enrichment, burger samples were plated onto polymyxin - acriflavine - lithium chloride - ceftazidime - aesculin - mannitol (PALCAM) agar (Conda, Madrid, Spain), Baird Parker agar (Oxoid Ltd., Hampshire, UK) and* Salmonella*-*Shigella* (SS) agar (Conda, Madrid, Spain) for the detection of* L. monocytogenes*,* S. aureus *and* Salmonella-Shigella*, respectively. Three colonies from each plate were selected for biochemical characterization. Biochemical identification of the organisms being under study was made using the method described by Barrow and Feltham [34].* L. monocytogenes*,* Salmonella* and* Shigella* colonies were biochemically identified using API* Listeria* and API 20E test kits (BioMerieux Inc., Lyon, France), respectively. The biochemical tests used to confirm* S. aureus *were coagulase test, catalase test, indole production, methyl red test, Voges-proskauer reaction, urease production, citrate utilization and sugar fermentation. The existence of coliforms and* E. coli* in samples of burgers was also tested using the most probable number (MPN) method [33]. First, mold and yeasts were detected and enumerated by diluting 1 g of burger sample in 9 mL of 0.1% peptonate water (Conda, Madrid, Spain), then, they were let for a two minute of homogenization. Subsequently, serial dilutions were made using 0.1% peptonate water. After that, samples were plated, using potato dextrose agar (Conda, Madrid, Spain) acidified to pH 3.5 with 10% tartaric acid solution. Next, all plates were incubated at 25°C and colonies were counted after 72 h. The results were expressed as CFU/g.

### 2.7. Statistical Analysis

All analyses were done in triplicates. Values were expressed as mean ± SD. One-way ANOVA and Duncan's multiple comparison tests were applied to compare the results with statistically significant differences for p values (*P *< 0.05). Version 19 of the IBM SPSS statistics software (IBM Corp., Armonk, NY, USA) was used to perform all statistical analysis.

## 3. Results and Discussion

### 3.1. Physical and Chemical Characterization of Algae Powders

**Figure 1:**
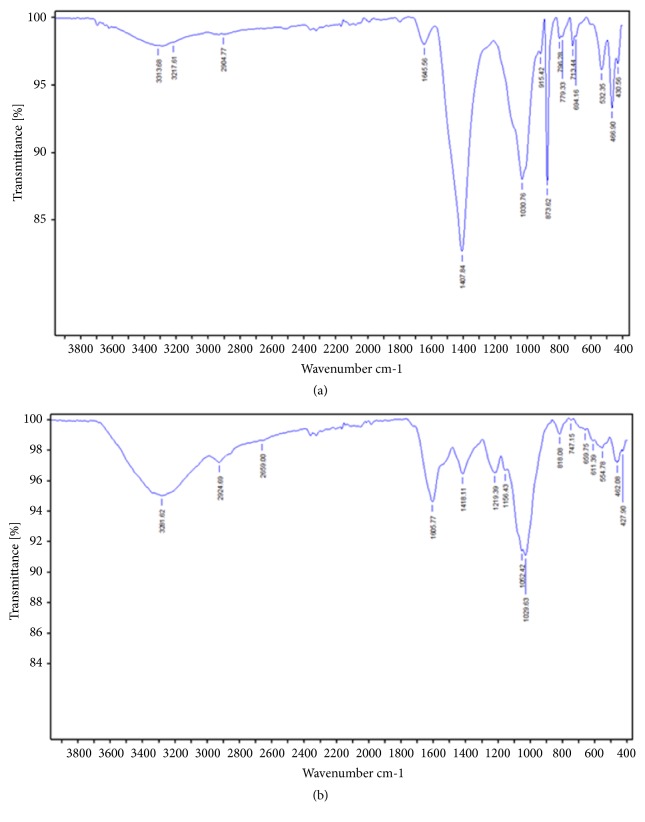
FT-IR spectra of the powder of (a)* C. compressa* (brown algae) and (b)* J. adhaerens* (red algae).

The compositions of algae (*C. compressa* and* J. adhaerens*) powders have been analyzed and the results are presented in Table 1. The crude fat and proteins contents were around 2.8% and 9.9%, respectively, irrespective of the type of algae. Ash content was high, 39.56%, in* C. compressa*, while* J. adhaerens* had the lowest ash content (36.83%) and the highest moisture (12.64%). Total dietary fiber (TDF) of algae ranged from 51.68% to 57.33%, with differences in the content of the insoluble and soluble dietary fiber fractions [35]. Powder algae (pH = (6.88-6.91); a_w_ = (0.35-0.41)) contained between 34.54 and 39.11% of total sugars, principally neutral, between 28.06 and 30.49%, with minor amounts of uronic acids between 4.05% and 11.05%. These chemical attributes were confirmed qualitatively by FT-IR analysis (Figure 1). The chemical content of the two studied algae was within the range reported by various authors for brown and red algae [35, 36]. Fibers and polysaccharides closely associated with cell wall proteins might also play a role in physicochemical properties of algae such as water-holding (9.57-13.82 g/g DW) and oil-absorption (2.2-3.8 g/g DW) capacities [1, 13], which suggests it might be able to stabilize food emulsion [36].

Brown seaweed (*C. compressa*) presented a variety of shades ranging from dark green to dark chestnut by way of yellowish-greens and masking the green of the chlorophyll with the existence of phycophin and xanthophyll where L^*∗*^ values were higher than b^*∗*^ values). However, the range of colorings from pink to red in* Jania* (red seaweed) is a result of the combination of chlorophyll, phycoerythrin and phycocyanin (high a^*∗*^ values) (Table 1). The pigments composition had a positive correlation with antioxidant activity as previously described [1, 13]. At 10 mg/mL, the ethanolic extract of* Cystoseira* powder exhibited higher DPPH scavenging activity (97.33%) that was higher than that for* Jania* powder (82.29%). A similar tendency was also observed for FRAP (Table 1). Due to their important biochemical characteristics (high content of dietary fiber and polysaccharides),* C. compressa* and* J. adhaerens* can potentially be used as natural ingredients to produce functional and nutritional food products and can be a healthy food source.

### 3.2. Technological Process

**Figure 2:**
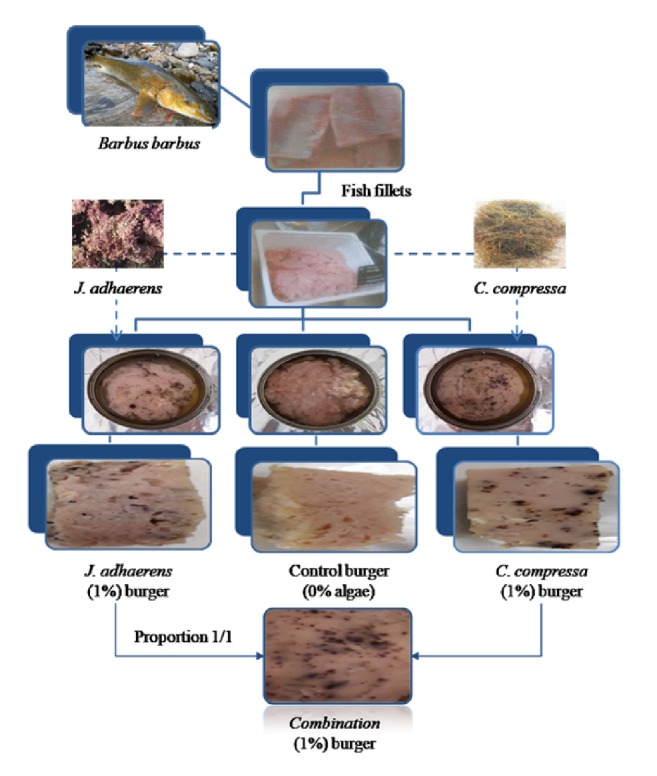
Diagram describing the main stages of the manufacturing process of common barbel fish burgers.

Actually, freshness highly contributes to the quality of fresh fish which is a greatly perishable product. The principal method to assess this parameter is sensory evaluation. The sensory evaluation method used in this study was the Quality Index Method (QIM) developed by the Tasmanian Food Research Unit [37]. According to this method, notes from 0 to 1; 0 to 2 or 0 to 3 demerit points (or indexes) were attributed to the changes in the smell, texture, appearance and color of the eyes, skin and gills of fish. The points attributed to barbel quality were summed up to give an overall sensory score or an overall quality index (IQ) equal to 6 (complete score 6/6). By comparing this value with the standard calibration curve, it was concluded that the raw material of this study reflected good sensory, microbiological and hygienic quality. In addition, this quality index provided a quantitative estimate of the shelf-life of this product with satisfactory precision. In the current study, fish are classified into 3 size categories (small, medium or large) based on their weight (Table 2). The yields we obtained during fish fillets preparation are illustrated by Table 2. The results showed a non-significant difference of yields for small or medium weight (*P *> 0.05) contrary to those obtained with the large one. Indeed, higher yields of fillets (44%) were obtained using large fish. These results could be explained by the difficulty of handling small fish. The yields of flesh, minced flesh and burgers were evaluated for the three fish categories. Hence, it was possible to transform a quantity of freshwater fish of 10 kg into around 3.6 kg of burger, including the ingredients added to the formulation, distributed among 36 cans (Figure 2).

### 3.3. Preliminary Results

Dried and ground* C. compressa* and* J. adhaerens* were added at 0.5, 1 and 1.5% (w/w) as dietary fiber and phycocolloids in the barbel burgers. Then the burgers were tested for their physicochemical, textural and sensory properties.

The results of these analyses are summarized in Table 3. Fish burgers without algae and containing the lower percentages of brown and red algae (0.5% and 1%, w/w) had the highest sensory scores (*P *< 0.05). These results were similar to those of Barkallah et al. [27] who revealed that fortified burgers with low* Spirulina* percentages had high sensory and textural qualities. The additions of high percentages of the two algae (1.5%) in the barbel burgers had the lowest levels of taste, color, flavor and general acceptability (*P *< 0.05). Furthermore, the existence of an inappropriate flavor, explained by the production of products promoting lipid oxidation as well as the production of metallic flavors by minerals from the algae has been noted. While, the algae powder macrostructure decreased (*P *< 0.05) the burger flavor scores for 1.5% (scores of 7.23 and 7.4), there was no significant difference for this parameter between control burgers and burgers supplemented with 1% of algae (*P* = 0.553 (*J. adhaerens*) and* P* = 0.999 (*C. compressa*)). The algal addition at several percentages enhanced texture scores (*P *< 0.05). Indeed, this improvement (*P *< 0.05) was mainly observed by adding* C. compressa *(0.5%) and* J. adhaerens* (1%) (scores of 8.19 and 8.16 instead of 7.96 for the control). Moreover, it was noticed that the fish burgers' color with the greatest percentage of algae (1.5%) changed from yellowish to a darker color and this was considered as an inappropriate sensory characteristic (bad appearance) by the panelists (*P *< 0.05). In addition, the burgers containing this percentage of algae had also the lowest scores of mouth tastes and overall acceptability (*P *< 0.05) for the presence of insoluble particles from algae. Generally, there was no important difference for these parameters between control burgers and those supplemented with lower quantities of algae. Fish burgers made with algae powder (1%) were found to be firmer and less greasy. It seems that the absence of greasy films observed for these burgers reinforced the sensory perception of the firmness as well as the improvement of the visual texture [1, 13]. The changes in texture were one of the most important parameters for assessing and addressing the difficulties found in functional foods [1, 28]. This key tool, strongly correlated with sensory properties, was used to determine the organoleptic quality of fish burgers [38]. The values of texture parameters (hardness, elasticity and cohesiveness) of control and burgers with different percentages of* J. adhaerens *and* C. compressa* are illustrated in Table 3. The addition of the two algae in all formulations increased the hardness and elasticity of burgers (*P *< 0.05). These results were similar to the work of Fernandez-Martin et al. [39] where the addition of algae (*Wakame* and* Nori*) in pork meat significantly improved its hardness and elasticity. The important hardness and elasticity values were mainly detected for burgers with 1% of* J. adhaerens* and* C. compressa* which had values of 8.12 N and 8.12 mm as well as 8.48 N and 7.21 mm, respectively. The cohesiveness of the control burger (0.24) was similar to that recorded in burgers containing 1% of* C. compressa *(0.25). This result differs from that published by Cofrades et al. [35] who evaluated the decrease in cohesiveness and resistance of gel/emulsion food systems after adding three edible algae (*H. elongata*,* U. Pinnatifida* and* P. umbilicalis*) in them. The higher amount of algae in burgers (1.5%) considerably affected (*P *< 0.05) the texture and rigidity of the final product (1.79 and 1.94) by disrupting the arrangement network of algal particles in fish burger [1, 13]. By contrast, the incorporation of 0.5% or 1% of the two algae in barbel burgers did not significantly change the cohesiveness recorded with the control. Hence, it was estimated that the supplementations of burgers with 1% of* C. compressa* or* J. adhaerens* both conserved and improved the textural characteristics and sensory acceptability of the final products already accepted by the panelists. Based on these sensorial and textural criteria, fish burgers enriched with 1% of algae were selected for further analyses so as to assess their functional, physicochemical microbiological and antioxidant properties. Added to that, the incorporation of a combination (1%, (w/w)) of* C. compressa*/*J. adhaerens *(proportions of 1/1) was prepared.

### 3.4. Nutritional Properties of Fish Burger

The moisture content, total solids, ash, protein and lipid contents of burgers are provided in Table 4. The chemical composition analysis of control burger showed its richness in water (77.57% in relation to the fresh weight). The total solids (TS) content was 22.43% (relative to fresh weight) and its consisted in 78.25% proteins, 8.34% lipids and 11.53% ashes. These results are in agreement with those found by Ben Atitallah et al. [1], Siddaiah et al. [40], and Vanitha et al. [41] for fish burgers made with* Cyprinus carpio*,* Hypophthalmichthys molitrix* and* Catla catla*. In this study, adding algae (1%, w/w) considerably improved the levels of the total solids (*P* < 0.05) and ash (*P* < 0.05). The decrease of proteins and lipids contents of burgers supplemented with algae compared to control seemed as if they would be non-significant (*P* > 0.05). This reduction confirmed the natural richness of freshwater fish in proteins and lipids [1, 13]. The highest values of total solids (*P *< 0.05) and ash (*P *< 0.05) were found in burgers supplemented with* C. compressa* (Table 4), showing the initial richness of brown algae in total solids and ash. Similarly, the combination of 1% (w/w)* C. compressa*/*J. adhaerens *(proportions of 1/1)) exerted a greater effect than that recorded after the sole addition of* J. adhaerens* (*P* < 0.05).

### 3.5. Chemical Composition and Antioxidant Potential of Fish Burgers

**Figure 3:**
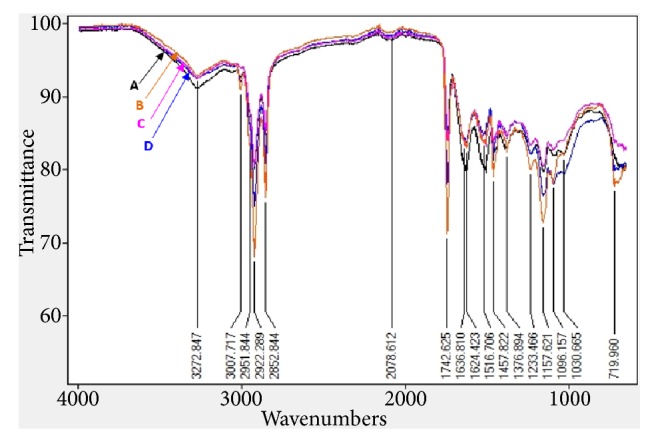
FT-IR analysis of (A) control burger, (B) 1%* C. compressa *burger, (C) 1%* J. adhaerens *burger, and (D) 1% combination burger.

These nutritional characteristics were qualitatively confirmed by FTIR analysis. The infrared spectrum of the control samples demonstrated some differences compared to those of the algae-supplemented burgers (Figure 3). The spectra showed a strong and wide absorption peak at 3272 cm^−1^ corresponding to the hydroxyl (O-H) stretching vibration of proteins, polysaccharides (such as glycogen from fish muscle and hydrocolloids from algae) and water. The strong absorption in the region of 3000 cm^−1^ and 2800 cm^−1^ proved the presence of the NH_2_ groups, which reflected the high content of protein in burgers. The bands at 1636 cm^−1^ (1590-1650 cm^−1^), corresponded to the (NH) and (C=O) groups of amides I, and the absorption band at 1516 cm^−1^ (between 1500 and 1560 cm^−1^) indicated the existence of (NH) groups and asymmetric (N=O) groups of amide II [42]. Lipids were characterized by the existence of an absorption peak at 1742 cm^−1^ suggesting the presence of ester groups (C=O) of hydrocolloids, while those at 1457 cm^−1^ and 1376 cm^−1^ mainly presented the C-OH stretching vibrations of carboxylic acids [28]. The spectrum of barbel burger supplemented with* C. compressa* (1%) revealed the presence of three characteristic bands at around 1030, 1100 and 1450 cm^−1^. These bands were attributed to guluronic, mannuronic units and sulfate groups (S=O), respectively, suggesting the presence of alginate and sulphated fucoidans described in literature as matrix polysaccharides of* Cystoseira* species [8].

The pigments rate, DPPH-radical scavenging activity and reducing power (FRAP) were determined to evaluate algae contribution to the antioxidant properties of the resulting fish burgers (Table 5). All ethanolic extracts of burgers showed an anti-DPPH and reducing power activities at a concentration of 10 mg/mL (Table 5). The incorporation of algae not only significantly improved chlorophylls (*P *< 0.05), carotenoids (*P *< 0.05), and free radical scavenging but also reduced iron (reducing power) levels. The extracts with* C. compressa* had both higher DPPH free radical scavenging activity and reducing power (*P *< 0.05) than those with* J. adhaerens*. These antioxidant activities were correlated with pigments and carotenoids equipment of the two algae (Table 5) [1, 13, 19]. Fish burgers supplemented with* J. adhaerens* that contained lower quantities of chlorophylls (a and b) had an antiradical activity (65.05%) and a reducing power (0.648) lower than those of burgers with* C. compressa* (88.29% and 0.837) (*P *< 0.05). The combination of the two algae had an important reinforcing effect on the DPPH free-radical scavenging (73.71%) and iron reducing power (0.642) activities compared to the burgers treated only with* J. adhaerens* (*P *< 0.05). These results seems to be similar to that in the studies carried out by Jónsdóttir et al. [12] and Wang et al. [43] revealing an anti-DPPH and reduction oxygen radical capacities of seafoods extracts containing* F. vesiculosus *(brown algae). Wang et al. [43] noted the strong antioxidant potential of phlorotannin from* F. vesiculosus* (polymeric phlorotannin-rich subfractions) to be used as a natural antioxidant supplement in fish muscle and fishery products. Cox and Abu-Ghannam [10] evaluated the effect of the addition of Sea Spaghetti as a source of antioxidants, dietary fiber, total phenols, and radical scavenging activity (DPPH) in cooked beef patties. The results showed that the antiradical activities of algae-enriched beef patties correlated with their polyphenol and pigment contents [1, 13, 44]. In their work, Andrade et al. [45] noted the important role of polyunsaturated fatty acids, phenolic compounds, chlorophylls and carotenoids in the improvement of the antioxidant potentials of food products. Moreover, the high antioxidant and iron reducing power could not be attributed only to the single class of compounds (polysaccharides, sterols, tannins, mannitol and carotenoids) but also to the synergistic effect between all these molecules [46].

### 3.6. Quality and Shelf-Life Measurements

#### 3.6.1. Physical and Functional Properties of Fish Burgers

Table 4 illustrated both the physical (a_w_ and pH) and functional (WHC, OHC and SWC) characteristics of control and algae-fortified fish burgers. The control burger a_w_ was estimated at 0.983 and 0.205 for fresh and dry conditions, respectively. The addition of algae powder in burgers significantly decreased the values of a_w_ of fresh and dry burgers (*P *< 0.05). The burger supplemented with the alga having the highest fiber content (*C. compressa*) had the lowest a_w_. These results were in accordance with those reported by Jridi et al. [28], who highlighted an important decrease in the a_w_ of dairy desserts prepared with Allig variety highly rich in fibers compared to other varieties of dates. The combination of the two algae in a same burger improved the qualities of finished products (*P* = 0.01) as shown by the weak level of a_w_ of this burger. This result could be explained by the effect of the fiber combination and might contribute to maintain a good microbiological quality. The pH values of different formulations indicated that the control burger presented higher pH values than that of algae supplemented one (7.14). The addition of* C. compressa* (alone or in a mix with* J. adhaerens*) in burgers decreased this parameter (*P *< 0.05) by making it closer to neutrality (7.04 and 7.05). This very decrease might be attributed to the acid nature of algae which was added to the formulations (pH 6).

Water holding capacity and oil absorption property mean the ability to associate with water and oil, respectively [36]. As illustrated in Table 4, the addition of the two algae at (1%) significantly increased (*P *< 0.05) the WHC, OHC and SWC values of fish burgers compared to controls, highlighting its richness of texturing compounds that play an important role in improving the food texture stability throughout the storage period [28]. These results were similar to those obtained by Ben Atitallah et al. [1, 13], Cox & Abu-Ghannam [10], and Senthil et al. [43]. In their studies, Cox & Abu-Ghannam [10], López-López et al. [47], and López-López et al. [48] have shown that the addition of the edible* Himanthalia elongata* (Sea Spaghetti) in beef and hog products improved their water and oil retention capacities. Our study demonstrated that fish burger supplemented with 1% of* C. compressa* had higher SWC values (*P* = 0.0125) than that enriched with 1% of* J. adhaerens*. Considering WHC and OHC of these two burgers, no significant differences were measured (*P *> 0.05). These WHC and OHC were mainly attributed to the existence of large amounts of algal polysaccharides, which were a potential source of soluble and insoluble dietary fibers [44]. Insoluble fibers can influence the texture of foods not only because of their ability to absorb and maintain water but also due to their swelling characteristics. Indeed, these insoluble compounds can improve the coherence of meat products by forming an insoluble three-dimensional network [49]. The level of fibers in* C. compressa* was significantly higher (*P* = 0.005) than that of* J. adhaerens*. The combination of algae (1% w/w) in burgers contributed to the formation of a more heterogeneous structure. So, there was a synergic effect of the two algae on the properties of our burger matrix [50].

#### 3.6.2. Color Analysis of Fish Burgers

Actually, color is one of the main parameters in determining the consumer acceptance of food products [35]. In the current study, it has been evaluated by two types of conventional and polar coordinates in fish burgers. The CIE-Lab color parameters (L^*∗*^, a^*∗*^ and b^*∗*^), chroma (C^*∗*^) and tint (shade) (h^*∗*^) of fish burgers are presented in Table 6. As the burgers were formulated under the same experimental conditions, the changes in color were only the consequence of algae addition. Cofrades et al. [35] noted that the formulation of food products has undergone changes in color of meat products depending on their water, fat and pigments contents. This was confirmed in this study where the color attributes (L^*∗*^, a^*∗*^, b^*∗*^, C^*∗*^ and h^*∗*^) were affected by the addition of brown and red algae to fish burgers. This difference in color depended mainly on the type of added marine algae. The supplementation of burger with* C. compressa *caused a significant and more pronounced decrease in coordinates (L^*∗*^, a^*∗*^, b^*∗*^) (*P *< 0.05). These results are similar to those obtained during the color analyses of fish burger mixed with* Eucheuma *[51]. The fish burgers with 1% of* J. adhaerens *had both the highest L^*∗*^ and b^*∗*^ values among the algal samples (*P *< 0.05) and the most important h^*∗*^ values (*P *< 0.05), which indicated that their color tended to be red (a^*∗*^ = 5.62). In contrast, the fish burgers enriched with 1% of* C. compressa* tended to decrease in the red color (a^*∗*^ = 5.2) compared to control (*P *< 0.05). The color of burger supplemented with a combination of the two algae did not significantly differ from that of burger treated with* C. compressa* (*P *> 0.05). Based on these findings, it might be concluded that the addition of these two algae significantly reduced the redness of fish burgers (*P *< 0.05), which suggested that the color of the final food product shifted from dark red to light red (red algae) or green (brown algae). This close relationship between redness and the type of the added algae in food was previously observed with Sea Spaghetti and Nori seaweeds by Cofrades et al. [35]. Moreover, Moroney et al. [9] studied the effect of adding algae extracts containing polysaccharides on the decrease in redness on the surface of the patties of fresh pigs. In the current study, all colors of fish burgers with algae were different from those found in the* Wakame* algal samples [35] where differences in a^*∗*^ values were not important (*P *> 0.05). The research carried out by Jiménez-Colmenero et al. [50] showed that the addition of Seafood Spaghetti/konjac gel caused a decrease in L^*∗*^ and a^*∗*^ values and an increase in the yellow hue b^*∗*^ of frankfurters. Similarly, Kim et al. [52] published the effect of algae incorporation (*Laminaria japonica*) on fresh pork hams and the decrease of L^*∗*^ and a^*∗*^ values. In contrast, Sasaki et al. [11] proved that on the one hand fucoxanthin reduced the lightness value L^*∗*^ and on the other hand it improved the values of a^*∗*^ and b^*∗*^ of ground chicken breast meat. These algae pigments could be used as stable natural dyes for applications in the fish canning industry.

#### 3.6.3. Microbiology Quality Analysis of Fish Burgers

No mold,* Enterobacteriaceae, *yeast, coliform bacteria or foodborne pathogens (*Salmonella* spp.,* Shigella* spp.,* L. monocytogenes*,* B. cereus*, and* Campylobacter *spp.) were detected in any of the canned burgers during two months of storage at 4°C [1]. The absence of these pathogens suggested that the fish burgers were clean and safe even after a storage period. These microbiological results suggest that the production of the final fish product was carried out with good hygienic and sanitary practices. Note that these results have also been reported in numerous studies [1, 13, 43].

## 4. Conclusions

Algae were effective natural additives to canned fish burgers. In addition to bringing nutritional components, algae might be a suitable source of beneficial natural flavoring and coloring agents. Furthermore, algae, rich in dietary fibers, maintained the texture of the final product by improving its functional properties (water and oil holding capacities). The addition of algae significantly improved both the physicochemical composition and the organoleptic acceptability of the final fish products without alteration of their microbiological quality. These algae treatments not only improved the nutritional content of the prepared fish products but also increased their antioxidant action. All these results could be used to potentially produce a canned fish burger prepared from minced flesh of common barbel enriched with algae as a natural source of bioactive substances (chlorophylls and carotenoids). The reasonable selection of these algae as fortifier agents in fish based products appears to be considerable, as it improves the healthfulness of foods.

## Figures and Tables

**Table 1:** The composition analysis of algae powder.

Items	*C. compressa*	*J. adhaerens*
*Physical and functional properties*		
pH	6.91 ± 0.02	6.88 ± 0.01
a_w_	0.35 ± 0.01	0.41 ± 0.02
L^*∗*^	35.71 ± 0.84	36.54 ± 0.18
a^*∗*^	3.85 ± 0.01	21.05 ± 0.21
b^*∗*^	11.74 ± 0.09	14.22 ± 0.23
WHC (g of water/g DW)	13.82 ± 0.52	9.57 ± 0.31
OHC (g of oil/g DW)	3.80 ± 0.26	2.20 ± 0.14
*Chemical properties*		
Moisture (%)	10.87 ± 0.13	12.64 ± 0.24
Total solids (%)	89.13 ± 0.13	87.35 ± 0.24
Protein (%)	9.98 ± 0.19	9.81 ± 0.15
Crude fat (%)	2.80 ± 0.45	2.76 ± 0.51
Ash (%)	39.56 ± 0.14	36.83 ± 0.28
Total dietary fiber (%)	57. 33 ± 2.05	51.68 ± 1.36
Total sugar (%)	39.11 ± 1.78	34.54 ± 1.15
Neutral sugar (%)	28.06 ± 0.51	30.49 ± 0.22
Uronic acids (%)	11.05 ± 0.55	4.05 ± 0.43
Chlorophyll a (mg/g DW)	16.78 ± 0.42	12.44 ± 0.32
Carotenoids (mg/g DW)	6.85 ± 0.19	4.51 ± 0.42
Chlorophyll b (mg/g DW)	0.92 ± 0.07	0.50 ± 0.01
Scavenging activity (%)^*∗*^	97.33 ± 2.45	82.29 ± 1.98
Reducing power^*∗*^	1.47 ± 0.12	0.88 ± 0.09

Values are expressed as means ± standard deviations of triplicates. ^*∗*^: the DPPH scavenging activity and the reducing power were evaluated at a samples concentration of 10 mg/mL.

**Table 2:** Yields of steps of burger preparation.

Barbel size	Category 1^*∗*^	Category 2^*∗*^	Category 3^*∗*^
*Yields (*%)			
After heading	79.88 ± 0.68	78.46 ± 1.76	78.48 ± 1.61^B,C^
After evisceration and flaking	56.35 ± 0.85	60.14 ± 0.25	65.31 ± 0.7^B,C^
After fileting	39.45 ± 0.15	39.99 ± 1.07	44.05 ± 0.67^B,C^
*Total yields of fish fillets transformation steps (*%)			
Total flesh yield	41.16 ± 1.92		
Minced flesh yields	37.56 ± 1.81		
Burger	34.52 ± 1.92		

^*∗*^: fish are classified into 3 size categories (small (730 ± 20 g), medium (1341 ± 60 g), or large (2110 ± 85 g)) based on their weight.

(%) All the yields of the different stages are calculated in grams of meat obtained after each stage with respect to the initial weight of fish.

Category 1 *versus* Category 2: ^A^*P* < 0.05.

Category 1 *versus* Category 3: ^B^*P* < 0.05.

Category 2 *versus* Category 3: ^C^*P* < 0.05.

**Table 3:** Textural and sensory properties of control and algae-supplemented burgers.

	Control burger	0.5%	1%	1.5%	0.5%	1%	1.5%
		*J. adhaerens* Burger	*J. adhaerens* burger	*J. adhaerens* burger	*C. compressa* burger	*C. compressa* burger	*C. compressa* burger
Taste	8.25	8.02^A,B^	7.90^A^	5.78^A,B^	8.06^A^	7.96^A^	6.04^A,C^
Flavor	8.08	8.04	8.01	7.23^A,B^	8.00	7.99	7.4^A,C^
Texture	7.96	8.02^B^	8.16^A^	8.10^A^	8.19^A,C^	8.04	8.02
Color	8.02	8.12	8.10	7.84^A,B^	8.04	8.04	7.76^A,C^
Overall acceptability	8.10	8.12^B^	8.00	7.78^A,B^	8.04	7.98	7.70^A,C^
Hardness (N)	6.69	7.49^A,B^	8.12^A^	7.86^A,B^	7.72^A,C^	8.48^A^	9.4^A,C^
Cohesiveness	0.24	0.19^B^	0.32	0.40^A^	0.29	0.25	0.27
Elasticity (mm)	5.63	5.98^A,B^	8.12^A^	8.64^A,B^	7.06^A^	7.21^A^	8.12^A,C^
Rigidity	3.50	2.63^A,B^	2.07^A^	1.79^A,B^	2.50^A,C^	2.64^A^	1.94^A,C^

Three fish burgers (100 g) were prepared for each formulation (control and with different concentrations of algae powder).

Control *versus J. adhaerens* and *C. compressa *burger (0.5%, 1%, and 1.5%): ^A^*P *< 0.05.

1% *J. adhaerens* burger *v*ersus (0.5 and 1.5%) *J. adhaerens* burger: ^B^*P *< 0.05.

1% *C. compressa* burger *v*ersus (0.5 and 1.5%) *C. compressa *burger: ^C^*P *< 0.05.

**Table 4:** Physicochemical and biochemical characterizations of control and algae-supplemented burgers (1%).

	Control burger	1% *J. adhaerens* burger	1% *C. compressa *burger	Combination burger
Moisture (% FW)	77.57 ± 0.32	76.89 ± 0.11^A,B^	76.55 ± 0.295^A^	76.87 ± 0.025^A,B^
Total solids (% FW)	22.43 ± 0.32	23.11 ± 0.11^A,B^	23.45 ± 0.295^A^	23.13 ± 0.025^A,B^
Protein (% DW)	78.25 ± 0.125	77.90 ± 0.055	77.55 ± 0.095	77.65 ± 0.2
Fat (% DW)	8.34 ± 0.085	7.97 ± 0.035	8.15 ± 0.10	8.35 ± 0.09
Ash (% DW)	11.53 ± 0.03	11.86 ± 0.105^A^	13.06 ± 0.097^A^	12.73 ± 0.165^A^
Total dietary fiber (g/100 g DW)^*∗*^	0.20 ± 0.01	0.36 ± 0.04^A,B^	0.62 ± 0.13^A^	0.50 ± 0.07^A,B^
Solid pH	7.14 ± 0.02	7.12 ± 0.005	7.04 ± 0.015^A^	7.05 ± 0.0^A^
a_w_ (wet)	0.983 ± 0.002	0.973 ± 0.002^A,B^	0.963 ± 0.002^A^	0.970 ± 0.001^A^
a_w_ (dry)	0.205 ± 0.005	0.198 ± 0.0^A^	0.195 ± 0.002^A^	0.192 ± 0.002
SWC (mL/g DW)^*∗*^	2.997 ± 0.023	3.365 ± 0.02^A,B^	3.56 ± 0.020^A^	3.435 ± 0.015 ^A^
WHC (g/g DW)^*∗*^	2.12 ± 0.03	2.61 ± 0.07^A^	2.62 ± 0.023^A^	2.59 ± 0.015^A^
OHC (g/g DW)^*∗*^	0.86 ± 0.0125	1.10 ± 0.02^A^	1.12 ± 0.025^A^	1.14 ± 0.02^A^

Three fish burgers (100 g) were prepared for each formulation (control and with different concentrations of algae powder).

Control *vs.* (1% *J. adhaerens, *1% *C. compressa* and 1% combination) burger:  ^A^*P *< 0.05.

1% *C. compressa* burger *vs*. (1% *J. adhaerens* and 1% combination) burger:  ^B^*P *< 0.05.

^*∗*^Results are expressed as means ± SD (n=3).

The (%) is calculated relative to a dry weight basis *(DW)* for lipid, protein and ash whereas the moisture content and total solids are expressed with respect to a fresh weight basis *(FW)*.

**Table 5:** Pigment equipment and antioxidant activity of fish burgers (with and without algae).

	Control burger	1%	1%	1%
		*J. adhaerens *Burger	*C. compressa* burger	Combination burger
*Chlorophyll a *	ND	11.44 ± 0.11^A,B^	16.92 ± 0.28^A^	12.25 ± 0.48^A^
(mg/100 g DW)				
*Carotenoids *	ND	4.43 ± 0.22^A^	6.95 ± 0.38^A^	6.17 ± 0.14^A^
(mg/100 g DW)				
*Chlorophyll b*	ND	0.25 ± 0.012^A,B^	0.874 ± 0.019^A^	0.64 ± 0.013^A,B^
(mg/100 g DW)				
*Scavenging activity* (%)^*∗*^	40.09	65.05^A,B^	88.29^A^	73.71^A,B^
*Reducing power* ^*∗*^	0.411	0.648^A,B^	0.837^A^	0.642^A,B^

Three fish burgers (100 g) were prepared for each formulation (control and with different concentrations of algae powder).

Control *versus*(1% *J. adhaerens*, 1% *C. compressa, *and 1% combination burger): ^A^*P* < 0.001.

1% *C. compressa* burger *versus* 1% *J. adhaerens *and 1% combination burger: ^B^*P* < 0.05.

^*∗*^: the scavenging activity of DPPH free radicals (%) and the reducing power (absorbance at 700 nm) were determined at a sample concentration of 10 mg/mL.

**Table 6:** Color analysis of different samples of fish burger powder by evaluating classical and polar coordinates.

	Control	1%	1%	1%
	Burger	*J. adhaerens* Burger	*C. compressa* burger	Combination burger
L^*∗*^	40.6 ± 0.05	38.71 ± 0.17^A^	33.28 ± 0.38^A^	34.42 ± 1.15^A^
a^*∗*^	8.18 ± 0.06	5.62 ± 0.08^A^	5.2 ± 0.07^A^	5.39 ± 0.11^A^
b^*∗*^	18.11 ± 0.47	14.32 ± 0.09^A^	9.73 ± 0.0^A^	9.53 ± 1.01^A^
h^*∗*^	65.69 ± 0.007	68.57 ± 0.002^A^	61.86 ± 0.006^A^	60.53 ± 0.04^A^
C^*∗*^	19.87 ± 0.45	15.38 ± 0.11^A^	11.03 ± 0.03^A^	10.95 ± 0.93^A^

Three fish burgers (100 g) were prepared for each formulation (control and with different concentrations of algae powder).

Control vs. (1% *J. adhaerens*, 1% *C. compressa* and 1% combination burger): ^A^*p* < 0.05. (L^*∗*^, b^*∗*^, and a^*∗*^) are classical coordinates; (L^*∗*^, C^*∗*^, and h^*∗*^) shows polar coordinates.

L^*∗*^ represents lightness, a^*∗*^ represents redness, b^*∗*^ represents yellowness.

## Data Availability

The data used to support the findings of this study are available from the corresponding author upon request.
